# Coherence of Parental Representations Following Therapy for Autistic Children

**DOI:** 10.1007/s10803-024-06252-2

**Published:** 2024-01-28

**Authors:** Nisha Vashi, Alaa Ibrahim, Ava Pouyandeh, Jonathan A. Weiss

**Affiliations:** Department of Psychology, York University, 4700 Keele Street, Toronto, ON M3J 1P3 Canada

**Keywords:** Autism, Parent-child relationship, Parental representations, Coherence, Mental health

## Abstract

Autistic children experience high rates of mental health challenges, and links have been found between child mental health and the parent-child relationship. As parents of autistic children are often actively involved in their child’s treatment, it is important to consider aspects of the parent-child relationship within this context. The present study investigated changes in a component of the parent-child relationship, the coherence of parental representations, following participation in a 10-week cognitive behavioural therapy intervention designed to address autistic children’s mental health challenges. Relationships were examined between coherence and child characteristics (i.e., autism symptoms, mental health), and associations with child treatment outcomes (i.e., mental health). Participants included 81 children (89% boys) aged 8 to 13 years and their parents (85% mothers) aged 35 to 54 years. Baseline levels of coherence were related to children’s mental health symptoms but not autism symptoms. Although there were no significant changes in overall coherence across therapy, subscale-level improvements (i.e., concern, acceptance) emerged. Changes in coherence across therapy were linked with children’s post-intervention behavioural symptoms and were approaching significance for internalizing problems, but were not associated with externalizing problems. It is critical to investigate factors that shape the coherence of parents’ representations of their children, as this may provide insight into potential targets for intervention. Ascertaining whether participation in therapy improves parental coherence, and consequently child treatment outcomes, can advocate for parent-involved therapy, which will ultimately benefit the well-being of autistic children.

Autistic children experience high rates of mental health challenges, such as internalizing problems (e.g., anxiety, depression) and externalizing problems (e.g., conduct problems, hyperactivity/inattention) (Rosen et al., 2018). Associations exist between autistic children’s mental health and autism symptoms, and the parent-child relationship. For example, the emotional quality of the parent-child relationship is bidirectionally linked with children’s emotional and behavioural problems (Hickey et al., 2020), and less severe child maladaptive behaviours are predictive of more positive mother-child relationships (Orsmond et al., 2006). Autism severity (comprised of difficulties with conventional social interaction and communication, and restricted interests and repetitive behaviours) has been shown to be related to different aspects of the parent-child relationship, such as parent-child closeness, parent-child communication, and levels of emotional expression between parents and their children (Beurkens et al., 2013; Hoffman et al., 2009).

Parental representations, a component of the parent-child relationship, may also be associated with child characteristics. These representations reflect the information processing rules that guide parents’ interpretations of their child’s feelings and behaviours (Benoit et al., 1997; Main et al., 1985). Parents’ representations shape their responsiveness towards their children, and are related to child mental health and behavioural problems in the general population (Davidov & Grusec, 2006) and in autistic children (Berliner et al., 2020). Studies that have examined links between parental representations and autism symptoms have demonstrated mixed findings. One study found that parents who reported greater autism severity based on the Diagnostic and Statistical Manual of Mental Disorders – Fifth Edition (DSM-5; American Psychiatric Association, 2013) criteria (i.e., mild, moderate, severe/profound) used fewer mental state descriptors to describe their child, reflecting less detailed articulations of their child’s mental states (e.g., wilful, opinionated, clever), desires and wishes, likes/dislikes, and emotions (Ansari et al., 2020). It is possible that the salience of a child’s autism diagnosis and related symptoms may compromise parents’ tendency to reflect upon their child’s unique mental characteristics. In contrast, Kirk and Sharma (2017) reported that higher autistic traits (operationalized as challenges with conventional social skills, attention switching, attention to detail, communication, and imagination) were not associated with parents’ ability to describe the mental states of their children. Instead, the authors suggested that the closeness between parents and their children, and parents’ own individual traits (e.g., their mental health), enabled them to provide more nuanced descriptions.

As parents of autistic children are often actively involved in their child’s treatment (van Steensel & Bögels, 2015), it is important to consider parental representations within this context. One study found that a four-week intervention aimed at improving parents’ ability to understand autistic children’s mental states resulted in improved child behavioural and emotional symptoms (Enav et al., 2019). It was suggested that increases in parent self-efficacy following the intervention were related to improvements in children’s mental health. Further qualitative research reveals that following participation in parent-involved therapy, improvements are reported by parents in the way they describe their child, such that they are better able to understand their child’s motivation, see the potential for a happy future for their child, focus on their child’s needs, and reflect on their child in ways beyond their autism-related symptoms (Thompson & McFerran, 2013).

Parents’ representations can be evaluated for their coherence, defined as the clarity, consistency, multidimensionality, and authenticity of their verbal narratives about their children (Main et al., 1985; Oppenheim, 2006). Coherence involves recognizing both the content of parents’ narratives as well as *how* parents speak about their children, and provides an indication of parents’ perceptions of their children’s thoughts, feelings, and behaviours. Parents with incoherent representations may process information regarding their child in a unidimensional or distorted way, overemphasizing their weaknesses, being overwhelmed with concern, exhibiting difficulties in perceiving their child as separate from themselves, or providing overly idealized or poorly integrated descriptions of their child. On the other hand, more coherent representations are undistorted, multifaceted, and well-integrated, including strengths as well as challenging aspects of their child’s characteristics. Parents with high levels of coherence tend to: (a) focus their narratives on their child, (b) provide elaborate and complex descriptions of their child, (c) see their child as a unique and separate individual from themselves, (d) accept their child’s needs and abilities, and display appropriate levels of concern for their child (Koren-Karie & Oppenheim, 2001; Sher-Censor et al., 2013). Studies often use some form of interviewing to determine the coherence of parents’ thoughts and feelings, such as through the Insightfulness Assessment (Oppenheim & Koren-Karie, 2013) and the Working Model of the Child Interview (Zeanah et al., 1994). Similarly, the Five-Minute Speech Sample procedure (FMSS; Magaña et al., 1986) has been used as a source content to rate coherence with parents of non-autistic (Sher-Censor et al., 2016) and autistic children (Sher-Censor et al., 2017). Parents are required to provide a brief description of their child using an open-ended format, with minimal prompts from the interviewer. Verbal content analysis of brief and open-ended approaches such as the FMSS can enable researchers to capture parents’ true internal emotions, thoughts, and attitudes about their child (Gottschalk & Gleser, 1969). The FMSS approach is one of the most commonly used methods to elicit an understanding of parents’ expressed emotion towards their child (e.g., criticism, concern), the emotional climate of the parent-child relationship, and family relational processes, and has been shown to be a valid procedure for predicting child outcomes across diverse contexts (Sher-Censor, 2015).

In the general population, coherence has been shown to be related to various aspects of child mental health, such as self-regulation (Rosenblum et al., 2002; Sher-Censor et al., 2016) and emotional and behavioural problems (Oppenheim et al., 2004), while in families of autistic children, coherence has been shown to be related to parent characteristics such as emotional availability (Sher-Censor et al., 2017) and parent sensitivity (Oppenheim et al., 2012). Understanding how to improve ways that parents can identify children’s feelings and behaviours can promote their self-efficacy and empower them to support their children in the treatment context (Enav et al., 2019). Moreover, ascertaining whether parent participation in therapy improves parental coherence can provide valuable insights for the utilization of parent-involved treatment approaches. This may benefit both children and parents, as parent involvement in therapy is a common component of evidenced-based CBT programs for autistic children (Reaven et al., 2012; Scarpa & Reyes, 2011; Wood et al., 2015).

## Present Study

To our knowledge, the present study is the first to examine whether there are changes in the coherence of parents’ representations following participation in a 10-week CBT intervention for autistic children, targeting children’s mental health problems. Three primary research questions were addressed. First, how do baseline parental coherence ratings relate to children’s mental health and autism symptoms? Second, is parent involvement in therapy associated with changes in the coherence of parents’ representations of their children? Lastly, is parental coherence associated with more positive child treatment outcomes (i.e., mental health)? We hypothesized that children with greater mental health challenges and autism severity would have lower baseline parental coherence ratings. Parent involvement in therapy was expected to be associated with improvements in the coherence of parents’ representations. Lastly, parental coherence was hypothesized to be associated with improved child treatment outcomes.

## Method

### Participants

Families participated in one of two randomized controlled trials involving a 10-week CBT intervention for child mental health problems. Participants were recruited from community advertisements, word of mouth, autism service newsletters, and website postings. Across the two larger trials, 217 participants were screened for eligibility, and 129 participants were included. Children were required to have a diagnosis of autism from a healthcare practitioner in Ontario (e.g., pediatrician, psychiatrist, registered psychologist), and meet clinical cut-offs on either the Social Responsiveness Scale, Second Edition (SRS-2 Total T-Score cut-off > 59; Constantino & Gruber, 2012) or the Social Communication Questionnaire, Lifetime Version (SCQ-L Total Score cut-off > 14; Rutter et al., 2003). For children that did not meet cutoffs on the SRS-2 or SCQ-L (*n* = 2), research assistants administered the Autism Diagnostic Observation Schedule, Second Edition (ADOS-2; Lord et al., 2008) to confirm the autism diagnosis. Following this, one participant was removed because their diagnosis could not be confirmed. Children were also required to have parent-reported emotion regulation difficulties (e.g., anger, sadness, anxiety) or clinical problems associated with emotion regulation, as assessed with the Anxiety Disorders Interview Schedule for DSM-IV: Parent Version (ADIS-P; Silverman & Albano, 1996) or the Behaviour Assessment Scale for Children, Second Edition (BASC-2; Reynolds & Kamphaus, 2004)/Third Edition (BASC-3; Reynolds & Kamphaus, 2015). Families were required to attend all pre- and post-intervention research appointments and 10 therapy sessions.

Families were excluded if children did not meet IQ cutoffs (i.e., FSIQ-2 score below 79) on the Vocabulary and Matrix Reasoning subscales of the Weschler Abbreviated Scale of Intelligence, Second Edition (WASI-II; Weschler), which has been confirmed to be a reliable estimate of intellectual functioning (Zhou & Raiford, 2011). Twenty-five participants were excluded for not meeting this inclusion criteria. In the current sample, included children had an FSIQ-2 score between 79 and 140 (*M* = 104.82, *SD* = 15.21). Families were also excluded if parents reported child aggressive or self-injurious behaviours that were a serious safety concern (*n* = 4), if they were currently receiving other treatment to address emotion regulation difficulties (*n* = 5), or for meeting multiple exclusion criteria or extraneous reasons (*n* = 11). Twenty-two families declined to participate in the intervention, and 20 families were lost to follow-up. Of the 129 eligible and included families, 93 participants completed the intervention, and reasons for not completing the intervention included a lack of motivation, withdrawal during the waiting period, and data collection challenges. Of this group, 12 participants were removed due to specific data collection challenges related to COVID-19 (i.e., no post-intervention speech sample). For the present study, 81 dyads were included. Children (89% boys) were aged 8 to 13 years (*M* = 9.60 years, *SD* = 1.38 years), and their parents (85% mothers) were aged 35 to 54 years (*M* = 43.84 years, *SD* = 4.41 years). Additional parent and child demographics are displayed in Table 1.

**Table 1:** Parent and child demographics

	n (%)
**Child ethnicity**	
White	56 (69)
Visible minority	16 (20)
Prefer not to disclose	9 (11)
**Parent ethnicity**	
White	57 (70)
Visible minority	16 (20)
Prefer not to disclose	8 (10)
**Parent education**	
Less than a bachelor’s degree	17 (21)
Bachelor’s degree or more	56 (69)
Prefer not to disclose	8 (10)
**Parent marital status**	
Single/divorced	7 (9)
Married/common law	73 (90)
Prefer not to disclose	1 (1)
**Annual family income**	
< $49,999	3 (4)
$50,000 – $99,999	17 (21)
$100,000 – $149,999	10 (12)
> $150,000	30 (37)
Prefer not to disclose	21 (26)

### Measures

#### Coherence

Coherence was evaluated using the Five Minute Speech Sample procedure (FMSS; Magaña et al., 1986), in which parents were asked to speak for five minutes about their child without them present. Researchers administered the following prompt to parents:

I’d like to hear your thoughts about [child’s name] in your own words and without my interrupting you with any questions or comments. When I ask you to begin, I’d like you to speak for five minutes, telling me what kind of person [child’s name] is and how the two of you get along together. After you have begun to speak, I prefer not to answer any questions. Are there any questions you would like to ask me before we begin?

Speech samples were audio-recorded and transcribed, and were evaluated for their coherence on various scales following a methodology developed by Sher-Censor et al. (2013). Based on scales adapted from the Insightfulness Assessment (Koren-Karie & Oppenheim, 2001), the six scales consisted of focus, elaboration, separateness, concern, acceptance, and complexity. Speech samples were rated on a continuous ordinal scale from 1 to 7 on each of the six aspects of coherence. An overall coherence score was independently coded, and higher scores reflected a more undistorted, multifaceted, and well-integrated portrayal of the child, while lower scores reflected a more distorted, one-sided, and poorly integrated portrayal of the child. This overall coherence score was dichotomized to maintain comparability with prior studies that have utilized the FMSS to characterize coherence (Sher-Censor et al., 2016, 2017). Overall coherence scores between 1 and 4 were coded as incoherent, and overall coherence scores between 5 and 7 were coded as coherent. The speech samples were coded by the first author (NV), a graduate student (AI), and a research assistant (AP), who underwent training administered by the author of the FMSS coding scheme (Sher-Censor et al., 2013). Coders were blind to participant IDs and whether transcripts were from pre- or post-intervention timepoints. However, 28% of the transcripts contained information indicating when the FMSS was administered (e.g., spoke about experience in the intervention). Interrater reliability (i.e., *ICC*) was established by all three coders coding 20% of the transcripts, and was calculated based on a two-way random effects model (Shrout & Fleiss, 1979). Coders met regularly during the coding process to discuss and resolve any discrepancies through consensus. Across prior studies of families in the general population and in the autism population, interrater reliability ranged from moderate (*ICC* = 0.53) to good (*ICC* = 0.86) for the individual subscales, good (*ICC* = 0.82) to excellent (*ICC* = 0.95) for the continuous coherence score, and strong (*Kappa* = 0.83) to almost perfect (*Kappa* = 1.00) for the dichotomous coherence score (Foley et al., 2019; Sher-Censor et al., 2013, 2016, 2017, 2018; Sher-Censor & Yates, 2015). For the present study, pre-intervention interrater reliability was excellent (*ICC* = 0.92 to 0.97) for the individual subscales, excellent (*ICC* = 0.98) for the continuous coherence score, and almost perfect (*Kappa* = 0.88) for the dichotomous coherence score. Post-intervention interrater reliability was excellent (*ICC* = 0.91 to 0.98) for the individual subscales, excellent (*ICC* = 0.96) for the continuous coherence score, and almost perfect (*Kappa* = 0.91) for the dichotomous coherence score. This coding system has been validated in prior studies in which the coherence of mothers’ FMSS was associated with fewer behavior problems in preschoolers (Sher-Censor & Yates, 2015), was related to more positive depictions of the parent-child relationship in preschoolers’ narratives (Sher-Censor et al., 2013), and predicted changes in adaptation from preschool to first grade among children with self-regulation difficulties (Sher-Censor et al., 2016).

#### Child Characteristics

Autism symptoms were assessed at baseline with the parent-report Social Responsiveness Scale, Second Edition (SRS-2; Constantino & Gruber, 2012). This 65-item measure assesses social functioning in five domains: Social Awareness, Social Cognition, Social Communication, Social Motivation, and Restricted Interests and Repetitive Behaviour (RRB). Items are rated on a Likert scale from 1 ‘not true’ to 4 ‘almost always true’, and higher scores indicate higher levels of social difficulties. In the present study, the Social Communication and Interaction (SCI; *M* = 72.65, *SD* = 8.57), comprised of the Social Awareness, Social Cognition, Social Communication, and Social Motivation subscales, and RRB (*M* = 74.05, *SD* = 9.68) T-Scores were analyzed. Within a normative sample, internal consistency ranged from *α* = 0.94 to 0.96, test-retest reliability of the original version of the measure (Social Responsiveness Scale; Constantino & Gruber, 2005) ranged from *r* = .88 to 0.95, and interrater reliability between parents and teachers on the school-age form was *ICC* = 0.77 (Bruni, 2014). Validity of the SRS-2 has also been established, with a study demonstrating that autistic children obtained higher scores than a control group (Bruni, 2014).

Child mental health was assessed pre- and post-intervention using the Internalizing Problems (i.e., anxiety, depression, somatization), Externalizing Problems (i.e., hyperactivity, aggression, conduct problems), and Behavioural Symptoms Index (i.e., hyperactivity, aggression, depression, atypicality, withdrawal, attention problems) T-Scores from the parent-report Behavior Assessment Scale for Children, Second Edition (BASC-2; Reynolds & Kamphaus, 2004)/Third Edition (BASC-3; Reynolds & Kamphaus, 2015). This measure assesses the behaviours and emotions of children and adolescents and has been frequently utilized in autism samples (Bradstreet et al., 2017; Zhou et al., 2020). Items are rated on a Likert scale from 1 ‘never’ to 4 ‘almost always’, with higher scores indicating greater mental health challenges. The BASC-2/BASC-3 contains strong psychometric properties, with high internal consistency, test-retest reliability, and moderate to high concurrent validity (Bradstreet et al., 2017; Reynolds & Kamphaus, 2004, 2015; Zhou et al., 2020).

#### Covariates

As previous studies have demonstrated associations between coherence and parent mental health (Oppenheim et al., 2012; Sher-Censor et al., 2017), parent mental health was included as a covariate in data analysis. Parent mental health was measured using the total score of the Depression Anxiety Stress Scale (DASS-21; Lovibond, 1995). This 21-item self-report measure has seven items per subscale (i.e., depression, anxiety, stress), and items are rated on a Likert scale from 0 ‘never’ to 3 ‘almost always’, with higher scores indicating greater mental health difficulties. The DASS-21 has demonstrated good to excellent internal consistency in a non-clinical sample of adults (*α* = 0.82 to 0.90 for the individual subscales, and *α* = 0.93 for the overall score; Henry & Crawford, 2005) and in a sample of adults with autistic children (*α* = 0.77 to 0.90 for the individual subscales, and *α* = 0.91 for overall the score; Maughan & Weiss, 2017).

### Procedure

#### Study Design

Interested families completed a phone screening and online survey, which included the SRS-2, to assess eligibility for the study. Eligible participants attended an in-person appointment, where informed consent and assent were obtained. Demographic information on the parents and children (e.g., age, gender, family characteristics) was also collected at this appointment. Children completed a readiness for therapy interview and researchers administered the WASI-II (Wechsler, 2011). Families then returned for a second visit within two weeks to complete other measures, including the FMSS and BASC-2/BASC-3. Following this appointment, families were randomized to either a treatment immediate (TI) or waitlist control (WLC) group. The TI group commenced therapy within a week of randomization, and parents of children in the WLC group were asked not to enroll their child in any other CBT therapy or therapy addressing emotion regulation for three months. The WLC group then commenced the delayed intervention. All measures were re-administered between 10 and 14 weeks after beginning the intervention to both the TI and WLC groups. For the purposes of the present study, the TI and WLC groups’ pre- and post-intervention data were combined. Families were reimbursed for parking or public transportation costs, and children received a prize following the completion of therapy and research appointments. The study was approved by the University Research Ethics Board.

#### Intervention

The intervention was a 10-week, manualized CBT intervention for autistic children experiencing mental health challenges (Secret Agent Society: Operation Regulation; Beaumont, 2013). Each session involved education from a therapist, practice of emotion regulation strategies, and planning for home and school activities. Emotion regulation skills were taught through various activities such as monitoring bodily clues and observing facial expressions, practicing relaxation and mindfulness techniques (e.g., controlled breathing, body scans), and learning about coping strategies (e.g., cognitive restructuring). Parents were encouraged to participate in the session activities and facilitate home and school activities. They were also provided with a workbook that summarized session content, included strategies for assisting with children’s behaviours, and contained tips for helping children complete home and school activities. Therapy was administered by graduate students who attended one full training day involving reviewing the manuals and observing videos. Previous studies provide further detail on the intervention and content of the individual sessions (Thomson et al., 2015; Weiss et al., 2018).

### Data Analysis

Only participants with both pre- and post-intervention FMSS data were included. Statistical assumptions were evaluated (e.g., distribution of scores, linearity, independence of observations, homoscedasticity, multicollinearity) and the dataset was inspected for outliers, and no major violations were observed. Bivariate correlations were computed at baseline for pre-intervention (T1) characteristics (i.e., child/parent age, child IQ, child/parent mental health, autism symptoms, overall and subscale coherence scores). For the dichotomous coherence score, Mann-Whitney U tests were performed, with coherence (i.e., incoherent versus coherent) as the grouping variable and T1 autism symptoms (i.e., SRS-2 RRB and SCI T-Scores) and T1 child mental health (i.e., Internalizing Problems, Externalizing Problems, and Behavioural Symptoms Index T-Scores) as separate dependent variables. A power analysis conducted in G*Power 3.1 (alpha of 0.05 and power of 0.80; Faul et al., 2009) and the effect size estimated from a previous study of the emotional availability of mothers of autistic children that categorized parent FMSS reports as incoherent versus coherent (Sher-Censor et al., 2017) suggested that our sample size could detect moderate to large effects. To test the hypothesis that there would be improvements in parental coherence following the intervention, Wilcoxon signed-rank tests were conducted for the subscale scores and the continuous coherence score. A power analysis (G*Power 3.1; alpha of 0.05 and power of 0.80; Faul et al., 2009) suggested that our sample size was sufficient to detect small effects using this approach. Effect sizes for the Mann-Whitney U and Wilcoxon signed-rank tests were calculated using *r* for ordinal data, as described by Fritz and colleagues (2012). For the dichotomous coherence score, a McNemar test was calculated to determine changes in the proportion of parents’ representations that were categorized as incoherent versus coherent before and after the intervention. Lastly, to test the hypothesis that improvements in parental coherence would be associated with improved child treatment outcomes, linear regression analyses were performed, with predictors including a T1 child mental health variable (e.g., Internalizing Problems), covariates (e.g., parent mental health, child/parent age, autism symptoms), and change in overall coherence, and the dependent variable being T2 scores in a child treatment outcome (i.e., Internalizing Problems, Externalizing Problems, Behavioural Symptoms). Power analysis indicated that moderate effects could be detected using linear regressions with an alpha of 0.05, sample size of 81, and six predictors. Although an alpha of 0.05 was used for all analyses, this threshold of significance is arbitrary and may not serve as an optimal indication of a practically or clinically significant effect (Hubbard & Lindsay, 2008), so the present study’s data analysis approach considered both alpha values and effect sizes when interpreting the results. Due to the small sample size and the limited research examining parental coherence within the context of therapy for autistic children, the potential practical significance and relevance of these findings justified the risk of potentially increasing the Type I error rate to minimize Type II error, and no adjustments were made to correct for multiple comparisons.

## Results

### Analysis 1: Baseline Associations

As shown in Table 2, child IQ and SCI scores were not related to subscale or overall coherence scores (all *p*’s > 0.10). Parent characteristics such as age and greater mental health challenges were correlated with subscale scores (i.e., lower acceptance, lower complexity, higher concern) with effect sizes in the small to moderate range. Greater parent mental health challenges were also moderately associated with lower overall coherence. Similar patterns were found with child mental health, such that higher internalizing problems, externalizing problems, and behavioural symptoms were linked with lower scores on acceptance, complexity, and overall coherence, with effect sizes in the small to moderate range.

**Table 2:** T1 Spearman correlations between parent and child characteristics and coherence

	Predictor variables								Dependent variables						
	2	3	4	5	6	7	8	9	10	11	12	13	14	15	16
1. Child age	− 0.08	0.48^***^	0.07	0.18	0.11	0.29^**^	0.05	0.06	0.20^+^	− 0.15	− 0.15	0.03	− 0.15	− 0.12	− 0.16
2. Child IQ		− 0.07	0.03	− 0.22^*^	− 0.07	0.04	− 0.03	− 0.08	0.002	− 0.09	0.06	0.12	− 0.06	0.02	− 0.12
3. Parent age			0.16	0.05	− 0.05	0.21	0.10	0.10	0.006	− 0.11	− 0.04	0.11	− 0.22^*^	− 0.22^*^	− 0.18
4. Parent mental health				0.29^*^	0.28^*^	0.29^**^	0.27^*^	0.35^**^	− 0.05	0.004	0.0004	0.20^+^	− 0.36^**^	− 0.49^***^	− 0.49^***^
5. SCI^a^					0.69^***^	0.39^***^	0.30^**^	0.51^***^	− 0.03	0.14	0.10	− 0.10	− 0.13	− 0.07	0.004
6. RRB^b^						0.37^***^	0.24^*^	0.46^***^	− 0.12	0.18	0.03	0.13	− 0.21^+^	− 0.08	− 0.17
7. Internalizing problems							0.49^***^	0.73^***^	− 0.03	− 0.002	− 0.16	0.13	− 0.35^**^	− 0.36^***^	− 0.35^**^
8. Externalizing problems								0.79^***^	0.007	− 0.07	− 0.17	0.07	− 0.24^*^	− 0.41^***^	− 0.34^**^
9. Behavioural symptoms									− 0.06	0.02	− 0.05	0.04	− 0.30^**^	− 0.39^***^	− 0.31^**^
10. Focus										− 0.004	− 0.05	− 0.04	0.006	0.09	0.10
11. Elaboration											0.05	− 0.01	− 0.05	0.16	0.06
12. Separateness												− 0.03	0.02	0.09	0.07
13. Concern													− 0.33^**^	− 0.29^**^	− 0.58^***^
14. Acceptance														0.61^***^	0.71^***^
15. Complexity															0.76^***^
16. Coherence^c^															

^a^ SCI = Social Communication and Interaction

^b^ RRB = Restricted Interests and Repetitive Behaviour

^c^ Analyses used continuous coherence variable

+*p* < .10; *p < .05; **p < .01; ***p < .001

Parents who were categorized as incoherent had children with greater internalizing problems (*M* = 64.02, *SD* = 12.34) than parents categorized as coherent (*M* = 55.92, *SD* = 9.87), with a small effect size, *Z* = -2.58, *p* = .01. Likewise, incoherent reports were associated with greater behavioural symptoms index scores (*M* = 70.61, *SD* = 9.55) than coherent reports (*M* = 66.00, *SD* = 8.95), with a small effect size, *Z* = -2.10, *p* = .04. Group differences in externalizing problems according to incoherent (*M* = 60.49, *SD* = 11.34) versus coherent reports (*M* = 55.71, *SD* = 8.36) showed a similar pattern, but were only approaching significance, *Z* = -1.88, *p* = .06. There were no group differences in autism symptoms (i.e., SCI and RRB) according to dichotomous coherence reports (all *p*’s > 0.10).

### Analysis 2: Changes in Coherence Following Therapy

To investigate whether there were improvements in aspects of parental coherence following the intervention, Wilcoxon signed-rank tests were conducted. As shown in Table 3, post-intervention concern subscale scores were lower (*Z* = -3.38, *p* < .001), and post-intervention acceptance scores (*Z* = 2.48, *p* = .01) were higher, than pre-intervention scores, with effect sizes in the small to moderate range. There were no differences in other subscale scores or overall coherence following the intervention (all *p*’s > 0.10). With regards to dichotomous ratings of coherence, approximately 30% (*n* = 24) of parents were rated as coherent at pre-intervention, and this increased to 43% (*n* = 35) at post-intervention, though results of the McNemar test revealed that changes did not meet a statistically significant cut point, *p* = .07. At the same time, there was a substantial degree of substitution across categories, where 10 of the original 24 participants who were rated as coherent prior to the intervention were rated as incoherent following the intervention, and 21 of the 57 participants who were rated as incoherent prior to the intervention were rated as coherent following the intervention. Of these 21 participants that did improve in coherence, 90% (*n* = 19) had lower levels of concern, 48% (*n* = 10) were more accepting, and 33% (*n* = 7) had more complex descriptions of their children.

**Table 3:** Wilcoxon signed-rank tests from pre- to post-intervention

	T1 mean (*SD*); *n* = 81	T2 mean *(SD*); *n* = 81	U	Z	Effect size* r*
Focus	6.69 (*0.63*)	6.73 (*0.71*)	216.50	0.32	0.04
Elaboration	6.67 (*0.69*)	6.67 (*0.92*)	150.00	0.38	0.04
Separateness	6.73 (*0.76*)	6.86 (*0.61*)	79.00	1.09	0.12
Concern	4.38 (*1.04*)	3.70 (*1.35*)	425.00***	−3.38	0.38
Acceptance	4.65 (*1.49*)	5.11 (*1.21*)	1244.00*	2.48	0.28
Complexity	4.46 (*1.30*)	4.53 (*1.23*)	570.00	0.61	0.07
Coherence^a^	4.12 (*0.90*)	4.26 (*0.91*)	611.00	1.11	0.12

^a^ Analyses used continuous coherence variable

*p < .05; ***p < .001

### Analysis 3: Associations with Child Treatment Outcomes

Regressions were conducted to test the hypothesis that improvements in coherence would be associated with improvements in child symptoms following the intervention. Table 4 depicts the results of linear regression analyses of the associations between changes in coherence (operationalized as change scores) and T2 child treatment outcomes, while controlling for T1 clinical symptoms and covariates. For internalizing problems, the overall model accounted for 67% of the variance in T2 internalizing problems, *F*(6, 72) = 24.84, *p* < .001, with change in coherence accounting for 1% of unique variance (*p* = .08). This pattern was stronger for behavioural symptoms, with the overall model predicting 72% of variance, *F*(6, 72) = 30.60, *p* < .001, and change in coherence accounting for 3% of unique variance in behavioural symptom improvement (*p* = .007). These links were not found for externalizing problems.

**Table 4:** Linear regressions between change in coherence and T2 child treatment outcomes

	B	SE	t	R	R^2^
**Internalizing problems**				0.82	0.67
Constant	−﻿ 3.53	10.81	− 0.33		
Child age	− 0.22	0.69	− 0.32		
Parent age	0.14	0.21	0.68		
T1 Internalizing problems	0.77***	0.08	9.79		
T1 Autism symptoms	0.10	0.11	0.94		
T1 Parent mental health	0.14	0.12	1.24		
Change in coherence^a^	−1.32^+^	0.76	−1.75		
**Externalizing problems**				0.89	0.79
Constant	3.80	8.59	0.44		
Child age	0.10	0.52	0.19		
Parent age	− 0.07	0.16	− 0.42		
T1 Externalizing problems	0.91***	0.06	15.00		
T1 Autism symptoms	0.001	0.08	0.01		
T1 Parent mental health	0.08	0.09	0.85		
Change in coherence^a^	− 0.71	0.57	−1.25		
**Behavioural symptoms**				0.85	0.72
Constant	6.60	9.06	0.67		
Child age	− 0.20	0.55	− 0.37		
Parent age	− 0.03	0.17	− 0.16		
T1 Behavioural symptoms	0.83***	0.08	10.27		
T1 Autism symptoms	0.04	0.09	0.44		
T1 Parent mental health	0.12	0.09	1.23		
Change in coherence^a^	−1.68**	0.60	−2.79		

^a^ Change in coherence was calculated using continuous coherence variable

^+^*p* < .10; **p < .01; ***p < .001

## Discussion

This study demonstrated the utility of the Five Minute Speech Sample procedure (FMSS; Magaña et al., 1986) to characterize coherence in families of autistic children participating in a 10-week CBT program for children’s mental health challenges (Secret Agent Society: Operation Regulation; Beaumont, 2013). Adopting this brief, low-burden coding scheme yielded a reliable indication of a nuanced aspect of parental representations, reflecting *how* parents spoke about their children (e.g., complexity, acceptance) rather than solely the content of their narratives. Compared to self-report (e.g., Positive Affect Index; Bengtson & Schrader, 1982) or interview-based (e.g., Camberwell Family Interview; Calam & Peters, 2006) measures of parental representations and the parent-child relationship, coherence captured using the FMSS procedure elicited additional objective, behavioural indicators of parents’ understanding of the needs and abilities of their children.

### Baseline Associations Between Coherence and Parent and Child Characteristics

As expected based on research with parents of non-autistic children (Davis et al., 2020; Rogosch et al., 2004), parent age was associated with higher acceptance and complexity, and greater parent mental health challenges were associated with higher concern, and lower acceptance, complexity, and overall coherence scores. Contrary to our hypothesis, but in line with Kirk and Sharma’s (2017) findings, autism symptoms (i.e., social communication and interaction, restricted interests and repetitive behaviours) were not associated with coherence. Within the present study, components of coherence, such as concern and acceptance, may have been expected to be related to the severity of children’s autism symptoms (e.g., social difficulties, restricted interests) as these behaviours are less socially acceptable and may impact children’s overall functioning (Kenworthy et al., 2009). Parents frequently spoke about the *effects* of their child’s autism-related symptoms, such as problems with social interactions contributing to their child’s social anxiety, or communication challenges contributing to their child’s behavioural problems, but did not talk about actual autism symptoms per se, and this may explain why the SRS-2 scores were unrelated to coherence ratings. Additionally, the families in the present study were seeking treatment primarily for their child’s mental health problems, so behaviours and symptoms related to their child’s autism diagnosis may not have been as salient within parents’ descriptions when using an open-ended approach like the FMSS.

Consistent with our hypothesis, greater mental health challenges were associated with more incoherent representations. Studies with non-autistic children have also demonstrated that higher child internalizing and externalizing behaviours are related to lower parental coherence (Sher-Censor et al., 2018; Sher-Censor & Yates, 2015). It is possible that in children with more marked mental health symptoms (e.g., behaviour problems, depression, anxiety), parents may have had difficulty speaking about multiple dimensions of their child’s behaviour in open-ended situations such as the FMSS, instead focusing specifically on their challenges or negative attributes. As such, this may have resulted in parents portraying a unidimensional representation of their children. This is consistent with prior research revealing that in children with mental health challenges, parents may be more likely to speak about their child’s negative attributes (e.g., concern, criticism; Cartwright et al., 2011). There may also be a bidirectional nature to the association between coherence and children’s mental health, though this goes beyond the methods used in the present study. According to attachment theory, parents’ representations are not only shaped by children’s behaviours, but also contribute to them (Bowlby, 1969). If parents have consistent, clear, and multidimensional representations of their children, they may be able to meet their needs more effectively. This was evidenced in a study of autistic children showing that coherent mothers were more emotionally available (i.e., able to accurately interpret their child’s signals, respond to their needs, and appropriately structure interactions with their child) than incoherent mothers (Sher-Censor et al., 2017). On the other hand, if children’s needs are not effectively met, they may internalize their problems (e.g., depression), or display external symptoms such as aggression or conduct problems to engage their parents’ attention (Bates & Bayles, 1988; Cassidy, 1994). As such, it can be useful to consider the mechanisms that may lead parental incoherence to contribute to children’s mental health symptom expression.

Children’s mental health challenges were also associated with specific aspects of coherence. At the subscale level, greater mental health challenges (i.e., internalizing problems, externalizing problems, behavioural symptoms) were associated with lower acceptance and complexity. The associations revealed between acceptance and children’s mental health reflects patterns seen in studies of expressed emotion (EE; Bader et al., 2015; Katz et al., 2014), which is a measure of parents’ feelings and attitudes expressed for their children (Magaña et al., 1986). High EE reflects high levels of criticism or emotional overinvolvement, low warmth, or a negative relationship (Benson et al., 2011). As parents of autistic children may often directly attend to and support their child’s behavioural problems (e.g., aggression), externalizing problems (e.g., conduct problems), and internalizing problems (e.g., anxiety), it is unsurprising that associations were revealed between lower acceptance and greater mental health challenges in the present study. Complexity refers to the extent to which parents can describe their child and their relationship in a comprehensive and balanced manner, including mostly positive, but also some negative aspects of their child’s behaviour. In children with mental health challenges, parents may speak more about the negative aspects of their child and their behaviour (Baker et al., 2019), thus resulting in less complex portrayals of their child and their relationship.

### Changes in Coherence Following Therapy

Contrary to our hypothesis, there were no group-level changes in overall coherence following the intervention. As parents were involved in the intervention as co-facilitators, it was expected that there may be improvements in parents’ understanding of the needs and abilities of their children, and in turn, the coherence of their representations. These null findings may have occurred because parental representations tend to remain stable over time (Aber et al., 1999; Benoit et al., 1997; Theran et al., 2005). The intervention’s brief and child-focused nature, which did not directly target changing parental representations, may have lacked the dose or instrumental activities required to substantially shift parents’ schemata held about their children. Another study of children participating in the same intervention did not reveal improvements in parents’ overall EE following participation in their child’s therapy (Maughan & Weiss, 2017), suggesting that a lack of change in parent reports pre- to post-intervention may not be entirely unsurprising. The literature on parent reports of treatment involving non-autistic children is more mixed, with some studies noting changes in parent criticism and emotional involvement following therapy (Gar & Hudson, 2009), and others reporting no change in the number of parents’ critical comments following therapy (Moskovich et al., 2017).

There are a number of methodological factors that may have contributed to this lack of change in coherence across therapy. Many participants were categorized as incoherent both at baseline (70%) and following the intervention (57%), and this may have occurred because of the stringent criteria of the coherence coding scheme (Sher-Censor et al., 2013). Coders were trained to initially assign a maximum possible score of 7 across the individual subscales and the overall coherence score, and subsequently deduct points to derive a final score. As such, one statement could have had the potential to change the categorization of a speech sample from coherent to incoherent. For example, with regards to acceptance, participants received a score of 5 if they were accepting of their child for the majority of the transcript, but expressed a minor remark indicating a lack of acceptance. However, two remarks of this nature would earn a score of 4, and the transcript would be categorized as incoherent. In addition to this, there was a substantial degree of substitution regarding participants categorized as incoherent versus coherent prior to and after the intervention. Regardless, of the participants that did improve in coherence following the intervention (26%), the majority had lower levels of concern (90%), and many were more accepting (48%) and had more complex (33%) descriptions of their children compared to their pre-intervention FMSS. Past research on changes in parent descriptions following interventions for autistic children has indicated that parents tend to report a greater number of positive comments about their child post-intervention (Maughan & Weiss, 2017), reflecting higher levels of acceptance, are more likely to describe their children beyond the ‘here-and-now’, and are less likely to make comparisons to ‘normality’, which may be reflective of greater acceptance and lower concern (Brezis et al., 2015). As a result of participating in their children’s therapy, parents’ descriptions may show less concern, more acceptance, and more complexity when they are better able to appraise and make meaning of their child’s disability (Bayat, 2007; Hastings & Taunt, 2002), and are more understanding of the needs and abilities of their children (Zimmer-Gembeck et al., 2019).

### Associations with Child Treatment Outcomes

Improvements in coherence from pre- to post-intervention did not uniquely predict improvements in children’s externalizing (e.g., hyperactivity, aggression, conduct problems) and internalizing (e.g., anxiety, depression, somatization) problems. It is important to note that children with aggressive and/or self-injurious behaviours were excluded from the intervention, which may limit the variability of externalizing problems reported in the present study’s sample, and consequently may have contributed to the lack of an association observed between changes in coherence and changes in externalizing problems. On the other hand, improvements in coherence uniquely predicted improvements in children’s broad demonstration of overall behavioural symptoms following completion of the program. The Behavioural Symptoms Index on the BASC estimates the general level of functioning or presence of impairment (Reynolds & Kamphaus, 2015). This index includes items from the hyperactivity, aggression, depression, atypicality, withdrawal, and attention problems subscales, and is noted in the BASC manual to reflect the overall level of problem behaviour of a child. Given that the current intervention primarily addresses emotion regulation (Beaumont, 2013), which has been noted to be a transdiagnostic process (Aldao et al., 2010; Weiss et al., 2018), it may be better suited to address these holistic indicators of functioning, rather than symptom-specific change (i.e., internalizing or externalizing problems). As such, parents may be more reflective of their children in an omnibus manner, as improvements may be observed across multiple domains of functioning, and once parents have a better understanding of the needs and abilities of their children, they may be better able to promote the generalization of skills across multiple contexts (Shalev et al., 2020). Regardless of the directionality of the link between parental coherence and child treatment outcomes, these findings advocate for parent-involved therapy, as it has the potential to enhance overall treatment effectiveness (Silverman & Berman, 2000). Practitioners may conceptualize intervention programming for autistic children to not only address child symptoms, but also parents’ representations of their children. These findings also underscore the importance of designing and implementing holistic, transdiagnostic treatment goals when working with these families (Fusar-Poli et al., 2019), as it may impact both children and parents. This is especially relevant for autistic children, who may exhibit an array of transdiagnostic difficulties (e.g., emotion dysregulation), in addition to their core challenges central to the diagnosis.

### Limitations and Future Research Directions

There are a number of limitations to consider in this research. The majority of parents identified as White and were mothers, most of the children were males, families had relatively high income and education, and parents were motivated to seek treatment, and these factors have been linked to aspects of the caregiving experience and parents’ ability to respond effectively to the needs of their children (Evans et al., 2008; Gülseven et al., 2018). In addition, none of the autistic children had an intellectual disability, which raises questions about the generalizability of the study findings. In addition, the distribution of IQ scores was skewed in the present sample (i.e., platykurtic distribution), which may be partially attributable to the measurement of intellectual functioning using the two-subtest score on the WASI-II. With regards to the measurement of parental coherence, there was little variability in some of the coherence subscales, including focus, elaboration, and separateness, limiting the interpretability of the overall coherence score, which was largely variable because of the concern, acceptance, and complexity subscales. Additionally, as children with aggressive or self-injurious behaviours were excluded from the intervention, the generalizability of the present study’s findings to autistic children with more externalizing problems is limited. Finally, as improvements in coherence accounted for only a small amount of variability in improvements in behavioural symptoms following the intervention, it is important to consider additional variables that may be linked with changes in coherence. For example, including children with lower intellectual functioning (e.g., Ansari et al., 2020), children who did not meet cutoffs on the SCQ-L or SRS-2, and/or children with self-injurious or aggressive behaviours in the sample may have provided further insight into the additional symptoms and characteristics that may be linked with improvements in coherence following parents’ participation in their children’s therapy.

In terms of future directions, it would be valuable to capture the coherence of children’s representations of their relationship with their parents (e.g., Sher-Censor et al., 2013), and how this may be related to their own mental health symptoms. Research is also needed to examine potentially greater improvements in coherence within the context of parent-focused interventions, such as parent-child interaction therapy (Eyberg, 1988) or parenting interventions (e.g., The Incredible Years; Webster-Stratton, 2005). Future studies should continue to replicate and evaluate the fidelity of assessing the coherence of parental representations using the Five Minute Speech Sample procedure across different therapeutic approaches. It would also be interesting to examine the therapeutic processes (e.g., parent/child engagement, therapeutic alliance) that may be related to changes in parents’ representations following participation in therapy, as these common factors are associated with child treatment outcomes (Lambert & Barley, 2001). Lastly, given the brief structure of this intervention, future research may explore whether greater changes are observed in parental coherence and children’s symptoms following a longer intervention, and whether improvements are maintained over an extended period following therapy (e.g., 3 or 6 months following completion of the intervention).

## Conclusion

It is important to consider factors that shape parents’ representations of their autistic children, as these families face unique challenges (Bitsika & Sharpley, 2004; Hayes & Watson, 2013; Rosen et al., 2018). As coherence was associated with children’s internalizing problems, externalizing problems, and behavioural symptoms at baseline, these factors can be addressed within intervention programming to potentially support both children and parents. In addition, changes in coherence were associated specifically with improvements in children’s behavioural symptoms following the intervention, suggesting the potentially important role of parental coherence within the context of therapy in fostering improvements in children’s overall level of functioning. Finally, the present study ascertained that although parent participation in their child’s therapy did not directly improve overall coherence, there were improvements in parents’ level of concern and acceptance of their children. This finding serves to advocate for parent involvement in their child’s therapy, which will ultimately benefit the well-being of autistic children (Sofronoff et al., 2005).
